# Gene-based microsatellites for cassava (*Manihot esculenta *Crantz): prevalence, polymorphisms, and cross-taxa utility

**DOI:** 10.1186/1471-2229-9-118

**Published:** 2009-09-11

**Authors:** Adebola AJ Raji, James V Anderson, Olufisayo A Kolade, Chike D Ugwu, Alfred GO Dixon, Ivan L Ingelbrecht

**Affiliations:** 1 International Institute of Tropical Agriculture (IITA), Oyo Road, Ibadan, Nigeria; 2 USDA-Agricultural Research Service, Biosciences Research Laboratory, 1605 Albrecht Blvd., Fargo, ND 58105-5674, USA; 3 Africa Rice Center (WARDA), 01 BP 2031, Cotonou, Benin

## Abstract

**Background:**

Cassava (*Manihot esculenta *Crantz), a starchy root crop grown in tropical and subtropical climates, is the sixth most important crop in the world after wheat, rice, maize, potato and barley. The repertoire of simple sequence repeat (SSR) markers for cassava is limited and warrants a need for a larger number of polymorphic SSRs for germplasm characterization and breeding applications.

**Results:**

A total of 846 putative microsatellites were identified *in silico *from an 8,577 cassava unigene set with an average density of one SSR every 7 kb. One hundred and ninety-two candidate SSRs were screened for polymorphism among a panel of cassava cultivars from Africa, Latin America and Asia, four wild *Manihot *species as well as two other important taxa in the Euphorbiaceae, leafy spurge (*Euphorbia esula*) and castor bean (*Ricinus communis*). Of 168 markers with clean amplification products, 124 (73.8%) displayed polymorphism based on high resolution agarose gels. Of 85 EST-SSR markers screened, 80 (94.1%) amplified alleles from one or more wild species (*M epruinosa, M glaziovii*, *M brachyandra*, *M tripartita*) whereas 13 (15.3%) amplified alleles from castor bean and 9 (10.6%) amplified alleles from leafy spurge; hence nearly all markers were transferable to wild relatives of *M esculenta *while only a fraction was transferable to the more distantly related taxa. In a subset of 20 EST-SSRs assessed by fluorescence-based genotyping the number of alleles per locus ranged from 2 to 10 with an average of 4.55 per locus. These markers had a polymorphism information content (PIC) from 0.19 to 0.75 with an average value of 0.55 and showed genetic relationships consistent with existing information on these genotypes.

**Conclusion:**

A set of 124 new, unique polymorphic EST-SSRs was developed and characterized which extends the repertoire of SSR markers for cultivated cassava and its wild relatives. The markers show high PIC values and therefore will be useful for cultivar identification, taxonomic studies, and genetic mapping. The study further shows that mining ESTs is a highly efficient strategy for polymorphism detection within the cultivated cassava gene pool.

## Background

Cassava, *Manihot esculenta *subsp *esculenta *Crantz (2n = 36), is a starchy root crop and the primary staple for over 800 million people worldwide [1]. Native to the Neotropics [2], cassava is now cultivated in tropical and subtropical regions in Asia, Latin America and Africa. In 2007, Africa was the world's largest producer with 118 million tons out of a global production of 228 million tons [3]. Cassava can grow under adverse climatic conditions with erratic rainfall and on marginal land under low intensity management [4]. Because of these attributes, cassava plays an essential food security role in Africa. In addition to food, cassava also has important industrial uses for the production of starch-based products, alcohol and animal feed [5,6].

Like most vegetatively propagated crops, cassava is highly heterozygous. Low rates of flowering and the long breeding cycle further render conventional breeding of cassava arduous [7]. Breeders have used controlled wide crosses with wild *Manihot *species for cassava improvement: for example, resistance to mosaic virus and bacterial blight has been successfully transferred from *Manihot glaziovii *to locally adapted cassava cultivars in Africa [8,9]. Because the genus *Manihot *is largely inter-fertile, wild species could further broaden the genetic base of cultivated cassava [10]. Natural hybridization between wild *Manihot *and cultivated cassava has also been reported [11].

Molecular marker technology is an efficient tool for the characterization and management of plant genetic resources and also in crop improvement programs. Among the different classes of molecular markers, microsatellites or simple sequence repeats (SSRs) are widely utilized for genetic diversity and genetic mapping because they are largely co-dominant, multi-allelic, have good genome coverage and can be multiplexed on semi-automated systems [12,13]. In the case of cassava, SSR markers have been utilized for the characterization of genetic resources [14-16] and an SSR-based molecular genetic map for cassava comprising 100 markers was described [17]. Despite these advances, the development and utilization of molecular markers for cassava lags behind the achievements of other major crop plants. For example, in case of barley, a high density microsatellite consensus map with 775 SSR loci has been described [18].

In recent years, EST and cDNA resources for cassava have increased substantially [19-21]. Cassava genomic and EST resources will further vastly increase with the recent completion of a draft cassava genome sequence and associated EST projects . These datasets constitute valuable and inexpensive sources of molecular markers such as SNPs [22] and microsatellites, since these can be readily detected computationally. Thus, mining these resources provides an opportunity to greatly expand the database of molecular markers for cassava at minimal cost.

Cassava is a member of the Euphorbiaceae family. The invasive weed leafy spurge (*Euphorbia esula*) and the oilseed plant castor bean (*Ricinus communis*) are other important members of this family. A draft genomic sequence for castor bean is available and both castor bean and leafy spurge have extensive EST resources (; [23]). With the recent completion of the cassava genome sequence, cassava and castor bean will constitute a valuable comparative genomics system.

The objectives of this study were to (i) increase the number of SSR markers by mining a previously developed cassava 8,577 unigene set; (ii) characterize EST-derived microsatellite markers in terms of abundance and level of polymorphism; and (iii) assess their transferability to other taxa in the Euphorbiaceae. The approach taken has been to characterize and validate 192 microsatellites identified *in silico *from a cassava unigene set by DNA fingerprinting a sample set consisting of: cultivated cassava from Africa, Latin America and Asia; four wild *Manihot *relatives; and leafy spurge and castor bean, two more distantly related taxa within the Euphorbiaceae.

## Results and Discussion

### Frequency and distribution of microsatellites in the cassava transcriptome

A total of 18,177 ESTs were used in this study, which were clustered in 8,577 unigenes [20]. In total, 836 microsatellite repeats were identified in the 8,577 unigenes (Table 1). Perfect repeats and imperfect repeats, i.e. repeats with small insertions or deletions within the repeat, were identified. Mononucleotide repeats were not included due to difficulties in accurate sizing of polymorphisms. Of 836 EST-SSRs, 316 (37.8%) were perfect repeats while 520 (62.2%) were imperfect repeats. A total of 137 ESTs contained perfect as well as imperfect repeats. Also, several ESTs contained 2 or more perfect or imperfect repeats. Overall, 596 ESTs were identified in the 8,577 unigene set that contained microsatellite repeats.

**Table 1:** Frequency and distribution of different SSR types in the 8,577 cassava unigene set.


**SSR motif**	**Number of repeat units**												**Total**
		
	**5**	**6**	**7**	**8**	**9**	**10**	**11**	**12**	**13**	**14**	**15**	**≥16**	

AC	-		3	1			2		1				7
AG	-	7	50	20	12	7	8	4	1	3	1	1	114
AT	-	14	83	32	22	13	11	1	4	2	2	11	195
CG	-		9										9
CT	-	23	66	46	15	21	12	14	8	5	7	5	222
GT	-	4	10	6	4	2						1	27
AAC	1												1
AAG	7	5	21	7	4	2	2	1					49
AAT	3	2	2	3	1		1						12
ACC	1	2	4	1	2	1							11
ACG													0
ACT													0
AGC	6	5	6	1	1								19
AGG	3	1	3										7
AGT													0
ATC	4	3	3	7	1								18
ATG	4	2	8	4	1	1	1						21
ATT	11	3	11	1	5	1							32
CCG	1		2	1									4
CCT	1	1	2	5	1								10
CGG	2												2
CGT													0
CTG	2	2	3	1		1							9
CTT	8	6	15	3	10		2	1					45
GGT	3	1	6	3									13
GTT	1												1
AAAG		2											2
TTAT		1											1
TTTC			1										1
TCTT				1									1
AAAGA				1	1								2
TTTTG	1												1
													**836**

SSR loci were classified by repeat type and structure (Table 1). The most frequent number of repeats per motif was between 5 and 9, thus microsatellites in cassava ESTs are rather small, in agreement with previous findings in coffee [24] eggplant [25] and barley [26]. Since the unigene set represented 5.8 Mb of the cassava transcriptome, the average distance between SSRs is approximately 7.0 kb or one SSR-containing EST every 10.3 ESTs. This number corresponds well with barley where the average distance between SSRs was 6.3 kb or one SSR-containing EST every 13.3 ESTs [26], *Coffea *which has an average density of one microsatellite every 7.73 kb [27], but is lower compared to pepper (*Capsicum*) where one SSR was found in every 3.8 kb EST [28].

The highest proportion of repeat motifs comprised di-nucleotide (68.7%) repeats, followed by tri-nucleotide repeats (30.4%). Only five tetra- (0.6%) and three penta-nucleotides (0.3%) were found. This result is in contrast with the majority of earlier studies which report tri-nucleotide repeats as the most abundant class of repeats [28-31] but in agreement with studies in *Picea *species [32] and *Coffea *[24] where di-nucleotide repeats were found to be the most abundant class. Reported frequencies and distribution of EST-SSRs is variable for different plant and animal species. This can be partly attributed to the microsatellite search criteria and the characteristics of the EST database analyzed in these studies. Since the cassava EST dataset used in this study was sequenced from the 5'end, a greater proportion of the EST-SSRs could be located in the 5'untranslated region rather than in the coding region where tri-nucleotide repeats are expected to be the predominant repeat type.

EST-SSRs were identified in 9.7% of the total number of contigs in cassava, compared to 10.2% for pepper [28], 6.8% for coffee [27], and 7.5% for wheat [31]. In this study, the SSR search was conducted on a unigene set which will return non-redundant SSRs. This approach results in a realistic estimate of microsatellite repeat frequency and ensures that EST-SSR markers correspond to unique loci in the genome.

### EST-SSR marker development

Out of the 596 unique ESTs containing microsatellites, primer pairs were designed for 346; 167 with perfect repeats (48.3%) and 179 (51.7%) containing imperfect repeats. The remaining sequences were inappropriate for primer design mainly because of insufficient DNA sequence flanking the microsatellite core. In addition, duplicates (21) with an existing cassava SSR dataset [33] were eliminated. One hundred and ninety-two randomly chosen primer pairs flanking di-, tri-, or tetrameric SSRs were optimized for PCR amplification using one or two cassava genotypes, TMS30572 and/or TME3. Details of the primer sequences and expected product size with SSR motif are described [see Additional file 1]. Of the 192 primer pairs tested, 168 (87.5%) yielded scorable amplicons. This percentage of working primers (mono- and polymorphic) is in the range previously observed for zoysiagrass (1,044 out of 1,163 or 89.7%) [34], clementine (41 out of 48 or 85.4%) [35], eggplant (39 out of 50 or 78%) [25] and *Coffea *(18 out of 24 or 75%) [24]. Twenty-seven primer pairs produced weak or no amplicons leading to exclusion from further analysis. Nine microsatellites produced amplicons that were significantly longer than expected and most likely indicate the presence of introns. Five of these produced fragments larger than 500 bp and could not be scored accurately for small differences in fragment size. These were also excluded from further analysis.

**Figure 1:**
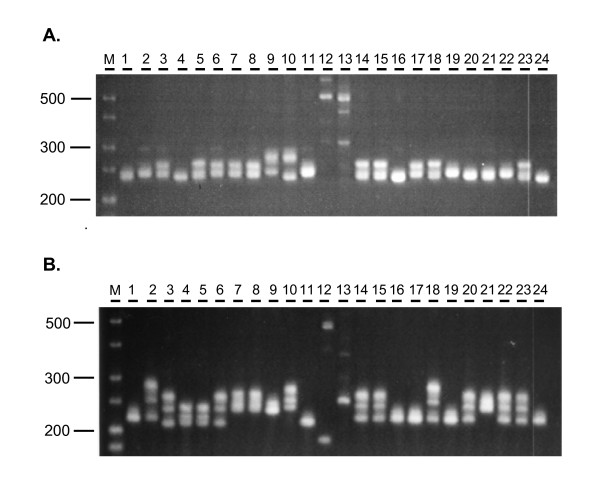
**Allelic variation of microsatellites MeESSR119 (A) and MeESSR106 (B) among genotypes of diversity panel 2 as detected on high resolution agarose gels**. Lanes: M is the size marker in bp; lane 1: Nachinyaya; 2: TME1738; 3: CM6740-7; 4: MCol1468; 5: MBra12; 6: MEcu72; 7: MPer183; 8: MTai18; 9: *M epruinosa*; 10: *M brachyandra*; 11: *M glaziovii*; 12: castor bean; 13: leafy spurge; 14: TMS30572; 15: TME419; 16: TME117; 17: TME3; 18: TME7; 19: TMS4(2)1425; 20: TME388; 21: TME1786; 22: TME530; 23: TME225; 24: TME638.

Next, primer pairs with scorable amplicons were assayed for polymorphism among cassava cultivars from Africa, Asia and Latin America (see Table 2). This panel included cassava landraces, elite breeding lines and some putative interspecific hybrids. To assess transferability of the EST-SSRs to other taxa within the Euphorbiaceae, the panel also included four wild *Manihot *species, castor bean and leafy spurge. In order to assess the potential of the newly developed EST-SSR markers for detecting polymorphism, all 168 primer pairs yielding PCR products were tested on this diversity panel or on a subset thereof, hereafter referred to as panel 2. Panel 2 is a subset of 24 genotypes, intended to represent the larger sample set; it includes cassava cultivars from 14 different countries in Africa, South America and Asia, three wild *Manihot *species, plus castor bean and leafy spurge (Table 2). Overall, 124 primer pairs (73.8%) showed polymorphisms in the germplasm analyzed, as illustrated for MeESSR119 (Figure 1A). Of the 124 polymorphic primers, 78 (62.9%) were for perfect SSRs while 46 (37.1%) were for imperfect SSRs. Thus perfect SSR repeats were more polymorphic than imperfect repeat types. When only cultivated cassava was considered, 40 out of 79 markers tested on panel 2 were polymorphic, corresponding to a frequency of 50.6%. The difference in level of polymorphism between cultivated cassava and all genotypes in panel 2 is due to the presence of additional, unique alleles in the wild *Manihot *species.

**Table 2:** *Manihot *and other Euphorbiaceae germplasm accessions with their origin screened for EST-SSR marker amplification and length polymorphisms.


**Genotype**	**Origin**	**Genotype**	**Origin**
	
***Manihot esculenta Crantz *cultivars (local name)**		***Manihot esculenta Crantz *cultivars (local name)**	
TME 1 (Antiota)	Nigeria	CM 1335-4	CIAT, Colombia
TME 3 (2nd Agric) ^1^	Nigeria	M Col 1468 (Mantequeira) ^1^	Brazil
TME 6 (Lapai-1)	Nigeria	M Bra 12 ^1^	Brazil
TME 7 (Oko Iyawo) ^1^	Nigeria	M Bra 383	Brazil
TME 9 (Olekanga)	Nigeria	M Bra 1045	Brazil
TME 14 (Abbey-Ife)	Nigeria	M Ecu 72 ^1^	Ecuador
TME 28 (MS-6)	Nigeria	M Per 183 ^1^	Peru
TME 117 (Isunikankinyan) ^1^	Nigeria	M TAI 18 ^1^	Thailand
TME 225 ^1^	Benin	IAC12	IAC, Brazil
TME 230 (Toma 36)	Togo	IAC14	IAC, Brazil
TME 232 (Toma 63)	Togo	Fecula Branca	Brazil
TME 279 (Obasanjo)	Nigeria	Branca de Santa Catarina	Brazil
TME 388 (Ewana Teraka) ^1^	Uganda		
TME 419 (Gbasekoute) ^1^	Togo	***Manihot esculenta *x* Manihot tristis***	
TME 530 (Mbundumali) ^1^	Malawi		
TME 638 (Ejura 79) ^1^	Ghana	TMS 00/0210	IITA, Nigeria
TME 568 (Mundele Paco)	Angola	TMS 00/0214	IITA, Nigeria
TME 1786 (Kibandameno)	Kenya		
Nachinyaya ^1^	Tanzania	**Wild relatives**	
TME1309 (Kigoma red)	Tanzania	*M epruinosa *^1^	Brazil
TME530 (Mbundumali)^1^	Malawi	*M tripartita*	Brazil
TME1738 (Buegwelu) ^1^	Zambia	*M brachyandra *^1^	Brazil
TMS 30572 ^1^	IITA, Nigeria	*M glaziovii *^1^	Brazil
TMS 4(2)1425 ^1^	IITA, Nigeria		
TMS 30555	IITA, Nigeria	**Euphorbiaceae**	
CM 6740-7 ^1^	CIAT, Colombia	*Ricinus communis *(castor bean) ^1^	USA
CM 523-7	CIAT, Colombia	*Euphorbia esula *(leafy spurge) ^1^	USA
CM 3306-4	CIAT, Colombia		

^1^accessions included in diversity panel 2 (see text for additional explanation)

Marker polymorphism for cultivated cassava in this study is about 2.7-fold higher compared to a study on cassava EST-SSRs where 18.4% polymorphism was detected (9 out of 49 primers tested were polymorphic) [36]. In citrus, more than 90% of EST-SSR markers were polymorphic which is higher compared to our results [35]. Fluctuations in level of polymorphism detected in EST-SSRs can be explained in part by the level of diversity among the germplasm surveyed. The higher frequency of polymorphism observed here compared to the study of Tangphatsornruang *et al*. [36] is possibly due to the inclusion of diverse landraces from 14 different countries in Africa, Latin America, Asia as well as elite cassava genotypes in this panel. Since polymorphism was scored on high resolution agarose gels, it is also possible that the level of polymorphism is higher than the frequency reported here since allelic variants with small size differences may not have been resolved on this system. Overall, our results show that mining ESTs for the development of SSRs is a highly effective strategy to increase the SSR database for cassava.

Out of 85 markers with clear amplicons tested on the 19 cassava cultivars of panel 2, 25 (29.4%) showed non-amplifying or null alleles in one or more genotypes. Therefore null alleles were common among the diverse cassava cultivars examined here. Null alleles for genic SSRs have also been described in other plant species such as for example kiwifruit [37], amongst others. The frequency of null alleles is influenced by several factors, including the diversity of the germplasm tested and technical aspects such as quality of the sequence data for primer development or PCR conditions. In a study on sunflower and related wild relatives, a clear relationship was found between genetic distance and amplification failures, with the frequency of null alleles ranging from 11.2% for an inbred sunflower accession to 34.5% among a wild sunflower species [38]. In a sample of wheat germplasm consisting of 52 diverse genotypes, null alleles for EST-SSRs occurred at a frequency of 45% [39] which is higher compared to the result obtained here. In view of the highly heterozygous nature of cassava and the occurrence of point mutations and/or insertion-deletions in the EST-SSR alleles outside the microsatellite repeat region (unpublished results), most null alleles observed in the present study are likely due to sequence variation in the primer binding sites.

SSRs are assumed to be locus specific. Cassava is considered an allotetraploid with disomic inheritance [40,41]. Therefore we expect amplification of one or two bands per locus. Out of 79 markers tested on panel 2, 49 (62%) amplified one or two alleles per locus while the remaining 30 (38%) markers amplified more than two bands for at least one genotype (see Figure 1B). In a previous study on genomic microsatellites in cassava [42], approximately 80% of the markers detected one or two alleles per accession, thus suggesting a fairly low degree of genomic microsatellite locus duplication. Our results show about a two-fold higher frequency of markers amplifying more than two bands per genotype. This is surprising in view of the fact that genic SSRs are generally considered to be more conserved compared to genomic SSRs [12]. However, multiple bands could also be due to the presence of multigene families. Therefore, in case of genic SSRs, the multiple bands could be due to cross-amplification of conserved members of multigene families. It should be noted that the frequency of multiple loci reported here represents a minimum estimate, considering that this result is based on high resolution agarose gel electrophoresis which does not resolve all allelic variants (unpublished observations). Similarly, the frequency of polymorphism reported here is a conservative estimate as markers with an allelic size range of 3-5 bp or less may have escaped detection. While the resolution of the agarose gel system is limited to about 3-5 bp, it does allow for a rapid screening of candidate microsatellite markers.

### Cross-species and cross-genera transferability

Cross-species transferability of the new cassava-derived SSR markers was tested for three wild *Manihot *species and two other Euphorbiaceae taxa; leafy spurge and castor bean. In general, the markers resulted in robust cross-species amplification with alleles of comparable sizes in cassava cultivars and the wild *Manihot *species. Out of 85 amplicons tested, 85 amplified for TMS30572 (100%); 82 for TME117 (97%); 80 for TME419 (94%) which are cassava cultivars, while 78 amplified for *M glaziovii *(91%); 74 for *M brachyandra *(87%) and 72 for *M epruinosa *(84.7%) which are wild *Manihot *species. Overall, a transferability of ~94% was observed for the wild *Manihot *relatives, i.e. 80 markers amplified alleles from one or more wild species. In agreement with this, it was previously demonstrated in cassava that genomic SSRs can be used to assess its wild relatives [14]. By contrast, only 13 (15%) and 9 (11%) respectively, of the EST-SSR markers yielded amplicons in castor bean and leafy spurge. Moreover, the sizes of the amplicons from castor bean and leafy spurge were often outside the allelic range observed in cultivated cassava and the wild *Manihot *species (see Figure 1), suggesting that these may not represent orthologous sequences. These numbers are very similar to those obtained in a study on the Compositae where transferability from sunflower (*Helianthus annuus*) to wild species was 88.6% while transferability to the more distantly related genera safflower (*Carthamus tinctorius*) and lettuce (*Lactuca sativa*) was 14.8% and 14.4% respectively [38]. Thus, the cassava EST-SSR markers display broad utility within the genus *Manihot *but limited utility beyond the genus level, in agreement with previous studies in plants [43-45]. It should be pointed out that amplification of the genic SSRs in the wild species does not necessarily imply that these loci are polymorphic in these species, since only a single accession was analyzed per taxon.

### Allelic polymorphism and genetic relationships

A subset of 20 randomly selected polymorphic EST-SSRs producing one or two amplicons per genotype was tested for their potential in genetic studies by ascertaining the genetic diversity in panel 2. Alleles were resolved by semi-automated fluorescence-based genotyping to attain a more accurate estimation of the allelic diversity and size range. Good amplification was obtained for all the markers across the tested genotypes. Leafy spurge and castor bean produced fewer amplicons compared to cassava and the wild *Manihot *species, in agreement with the results obtained from high resolution agarose gel electrophoresis.

**Figure 2:**
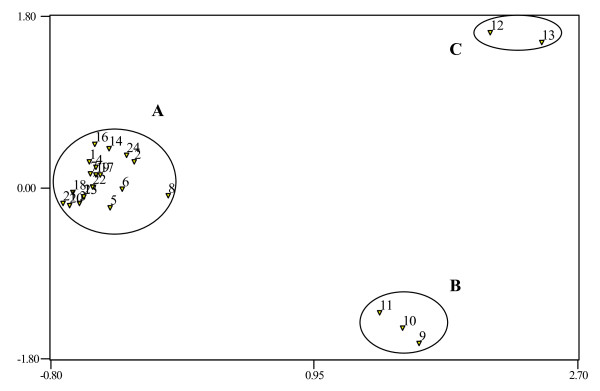
**Genetic differentiation by principal coordinate analysis showing distinct variation between cultivated cassava (A), wild *Manihot *species (B), castor bean and leafy spurge (C)**. The germplasm accessions are numbered according to the lanes in Figure 1.

**Figure 3:**
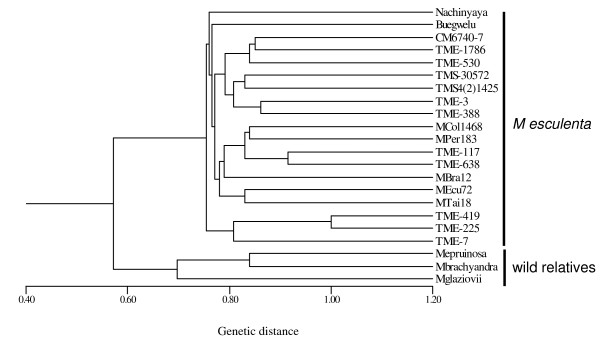
**Neighbor-joining tree showing relationships between 19 cassava cultivars and three wild *Manihot *species based on the allelic diversity generated using 20 new EST-SSR markers**.

The 20 polymorphic EST-SSR markers generated 94 alleles and discriminated all 24 accessions into different clusters according to their phylogenetic relationship. The principal coordinate plot (Figure 2) shows the genetic relationship and distribution of the panel. The first and second principal coordinates accounted for 60.79% of the total variation, and showed an effective clustering among the species. From the principal coordinate plot, three groups were clearly shown: (i) the cassava accessions (ii) the wild *Manihot *species and (iii) castor bean and leafy spurge. Next, a dendrogram was constructed to show the genetic differentiation of the cassava accessions and wild *Manihot *species (Figure 3). The dendrogram grouping showed the expected separation of the cultivated cassava accessions from their wild relatives, in agreement with the cluster grouping obtained from the principal coordinate plot. In addition, the dendrogram revealed expected genetic relationships between cassava cultivars based on their pedigree or previous diversity studies. For example, TMS 4(2)1425 and TMS 30572 are two progeny from TMS 58308 and group together in the dendrogram, in agreement with their shared ancestry [46]. Genetic similarity was also detected between TME225 and TME419, landraces from Benin and Togo respectively, in agreement with a previous genetic diversity study based on 18 genomic SSRs [47]. Overall, this analysis supports the botanical classification of the germplasm surveyed and suggests the usefulness of the EST-SSR markers for genetic diversity studies and other genotyping applications in cassava and its wild relatives.

The allelic diversity and extent of polymorphism for panel 2 accessions were also determined and are summarized in Table 3. The number of alleles per locus ranged from 2 to 10 with an average of 4.55 alleles per marker. The allele size of the original TME117 EST sequences all fall within the allele size range determined by fluorescence-based genotyping as summarized in Table 3, as expected. The number of alleles per locus is higher compared to a previous study on cassava EST-SSRs where this ranged from 2 to 4 [36]. In the latter study, alleles were detected on polyacrylamide gels after silver staining. The higher number of alleles observed in our study is likely due to the higher resolution of fluorescence-based genotyping as well as the inclusion of three wild species, leafy spurge and castor bean in the panel. In previous studies on genomic SSRs in cassava, an average of 8.6 alleles per locus [42] and 9.0 alleles per locus [15] was detected which is higher compared to the value 4.55 found here for the genic SSRs. The average allelic size range for the cassava EST-SSRs described here is 16.7 bp which is lower compared to the value of 25.4 for seven cassava genomic SSRs previously analyzed using fluorescence-based genotyping [42]. EST-SSR markers were found to be less polymorphic compared to genomic SSRs in other crop plants such as rice and barley [48,49]. In cultivated durum wheat, EST-SSR markers were less powerful in discriminating genotypes compared to genomic sources, though they produced higher quality markers, i.e. with fewer stutter bands [50]. The polymorphism information content (PIC) values of the EST-SSR markers identified here varied from 0.19 to 0.75 with an average of 0.55 (Table 3). These values are within the range of those reported for other plant species where average PIC values of EST-SSR markers varied from 0.32 to 0.66 (see [12] and references therein). In terms of allelic polymorphism, our results in cassava seem to support the findings in other species such as rice and maize where microsatellites from coding regions tended to have a narrower allele size range and be less polymorphic than those derived from genomic clones. It will be interesting to compare the EST-SSRs described here to the genomic SSRs previously developed for cassava in terms of discriminating power and quality.

**Table 3:** Simple sequence repeat fingerprinting results with number of alleles per locus, expected and observed allele size, and PIC values after analyzing germplasm accessions of diversity panel 2.


**Marker name**	**No alleles per locus**	**Size range**	**Expected size**	**PIC**

MeESSR8	4	155-170	166	0.57
MeESSR9	2	210-214	212	0.23
MeESSR10	4	128-157	154	0.62
MeESSR11	3	235-255	233	0.50
MeESSR15	8	147-180	157	0.70
MeESSR19	6	200-225	208	0.75
MeESSR22	3	169-185	183	0.53
MeESSR23	5	207-239	212	0.49
MeESSR26	5	243-254	247	0.76
MeESSR28	3	190-197	190	0.19
MeESSR29	5	139-152	150	0.72
MeESSR30	3	165-170	169	0.48
MeESSR31	4	188-202	201	0.59
MeESSR33	10	192-218	201	0.71
MeESSR60	7	154-173	165	0.66
MeESSR62	6	184-204	198	0.60
MeESSR63	3	182-200	199	0.49
MeESSR65	3	249-261	250	0.39
MeESSR66	4	220-229	229	0.65
MeESSR71	3	170-177	173	0.45
**Average**	**4.55**			**0.55**

### EST-SSRs as functional markers

To assess functionality of the new markers, BlastX searches were performed for 123 ESTs containing polymorphic SSR. These results are summarized [see Additional file 2] and showed that a putative function could be assigned to 50 ESTs (40.7%) assuming a threshold of E<1.10^-4^, while 54 (43.9%) corresponded to predicted or hypothetical proteins. Ten ESTs (8.1%) produced no significant match. Cassava ESTs that produced no specific matches, or matches to hypothetical or unknowns, were then compared to annotation of the original cassava EST-database  to identify potential Arabidopsis homologues. This search resulted in annotation of an additional 24 EST sequences, bringing the total number of matches to proteins with a putative function to 74 (60.2%). As expected, most searches retrieved sequences from castor bean (70) followed by poplar (22), two plant species whose complete genomic sequence has been determined and that are most closely related to cassava.

## Conclusion

In cassava, the number of published SSR markers is limited compared to other major crops. In this study, an in depth analysis of genic microsatellites in terms of density, level of polymorphism, and informativeness is presented. A new set of 124 genic SSR markers with PIC values comparable to genic SSRs in other plant species was developed. These resources will be of interest to the Euphorbiaceae research community in general and the cassava research community in particular, where research focuses on basic and applied aspects of cassava biology, including germplasm characterization, breeding applications, and phylogenetic studies of *Manihot *species. We have shown that mining ESTs is an effective strategy to identify functional microsatellites in the cultivated cassava gene pool with perfect repeats being more polymorphic that imperfect repeats. Nearly all genic SSRs are readily transferable to other species within *Manihot *but much less so with two other genera of the Euphorbiaceae examined here, *Ricinus communis *and *Euphorbia esula*.

## Methods

### Plant material

The cassava cultivars, wild *Manihot *relatives along with other *Manihot *accessions and castor bean summarized in Table 2 were collected from the Cassava Breeding Unit and the Genetic Resources Center of the International Institute for Tropical Agriculture, Ibadan, Nigeria. Leafy spurge DNA was provided by USDA-ARS, Fargo, ND, USA. DNA was extracted from leaf tissue as described [51].

### Searching for microsatellites and primer design

The identification and localization of microsatellites in ESTs was accomplished by a microsatellite search as described previously [52]. For *in silico *identification of the perfect repeats, the minimum number of repeat motifs was set at 6 for the di-nucleotide repeats and at 5 for all other repeats. For the imperfect repeats, the minimum number of repeats motifs was set at 7 for the di- and tri-nucleotide repeats and 6 for the tetra- and penta-nucleotide repeats. The repeats in Table 1 are the combined number of perfect and imperfect repeats; all possible combinations are listed for the di- and tri-nucleotide repeats while for the tetra- and penta-nucleotide repeats, only repeats for which microsatellites were retrieved are listed. The number of repeat units for the imperfect and perfect repeats separately can be obtained upon request. Primer pairs were designed using Primer3 . The major primer design parameters were set as follows: primer length from 18 to 28 (optimum 20), PCR product from 100 to 300 bp, optimum annealing temperature between 57 and 60°C. Primer sequences and annealing temperatures for polymorphic SSRs reported in this paper are given [see Additional file 1]. Information on additional primer pairs that failed to amplify, could not be easily scored or were monomorphic, can be provided upon request.

### Polymerase chain reaction conditions and product electrophoresis

PCR was performed in a 10 μl volume containing 10 ng DNA in 1.5 mM MgCl_2_, 1× PCR buffer (Bioline, United Kingdom), 0.2 mM of each dNTP, 0.2 μM of forward and reverse primer and 1.0 u Biotaq DNA polymerase (Bioline, United Kingdom). Reactions were performed in 96-well plates in MJ thermocyclers (PCT-100; MJ Research, Watertown, Mass, USA) under the following touchdown profile: (I) 3-min initial denaturation at 94°C; (II) 10 cycles of: 30 s at 94°C; 30 s at 60°C with 0.5°C decrease per cycle; 60 s at 72°C; (III) 25 cycles of: 30 s at 94°C, 30 s at 55°C and 1-min extension at 72°C; (IV) final extension was 5-min at 72°C. PCR reactions were optimized using 3 annealing temperatures: 55°C, 58°C and 60°C. Primer pairs that did not amplify under these conditions were subsequently tested using 1, 1.5 and 4 mM MgCl_2 _concentration for the 3 touchdown profiles. Success of amplification was checked on 2% agarose gels run in 1× TBE (89 mM Tris-Borate, 20 mM ethylenediaminetetraacetic acid) and stained with EtBr. Primer pairs with clean amplification products were then screened for polymorphism using the fingerprinting panels in Table 2. PCR products were run on 3.5% MetaPhor Agarose (Lonza, Rockland, ME, USA) or Super Fine Resolution Agarose (Amresco, Solon, OH, USA) in 1× TBE buffer and visualized by EtBr staining. Band sizing was estimated by comparison with the size marker HyperLadder V™ (Bioline, United Kingdom). A subset of polymorphic markers was resolved on ABI3100 DNA Genetic Analyzer and analyzed using GeneMapper v 3.7 (Applied Biosystems). The allelic data were used to determine the number and sizes of amplified alleles.

### Data analysis

Principal coordinate and cluster analyses were performed to estimate the discriminatory ability of 20 EST-SSR markers to assess inter and intra species relationships. Genetic similarity coefficient was generated from 94 EST-SSR alleles using the Nei and Li [53] method. The similarity matrix was used to construct a dendrogram using the neighbor joining method and the TREE VIEW sub program of the NTSYS-PC software [54]. Principal coordinate analysis was performed to show genetic relationships among the accessions. The polymorphism information content (PIC) of the markers was determined to measure the informativeness of the markers using the equation:



Where PIC*i *is the polymorphic information content of a marker *i*; P*ij *is the frequency of the *j*th pattern for marker *i *and the summation extends over *n *patterns [55,56]. For functional analysis, the translated EST sequences were searched against the GenBank non-redundant CDS translations + PDB + SwissProt + PIR + PRF excluding environmental samples from WGS project using the BLASTX algorithm (GenBank release May 2009; [57]).

## Authors' contributions

AAJR and OAK carried out all aspects of marker optimization and screening with contributions from CDU. AAJR and ILI were responsible for data analysis. ILI, AAJR, JVA, and AGOD co-wrote the manuscript. ILI conceived the study and provided overall coordination of the project. All authors critically reviewed the manuscript.

## Supplementary Material

Additional file 1 **Features of new set of polymorphic EST-SSR markers developed. **The data provided represent details of the new SSR markers e.g. marker name, primer sequence, PCR conditions, indication of perfect (P) or imperfect (I) repeat type, and expected product size. Click here for file

Additional file 2 **Identifiers of the polymorphic EST-SSR markers with their annotation following BlastX searches.** Click here for file
